# Autoimmunity as a sequela to obesity and systemic inflammation

**DOI:** 10.3389/fphys.2022.887702

**Published:** 2022-11-21

**Authors:** Victoria R. Kwiat, Gisienne Reis, Isela C. Valera, Kislay Parvatiyar, Michelle S. Parvatiyar

**Affiliations:** ^1^ Department of Nutrition and Integrative Physiology, The Florida State University, Tallahassee, FL, United States; ^2^ Department of Microbiology and Immunology, School of Medicine, Tulane University, New Orleans, LA, United States

**Keywords:** obesity, autoimmunity, inflammation, adipokines, metabolism

## Abstract

The rising prevalence of obesity presents a world-wide challenge as it is associated with numerous comorbidities including cardiovascular disease, insulin resistance and hypertension. Obesity-associated illnesses are estimated to cause nearly 4 million deaths globally per year, therefore there is a critical need to better understand associated pathogenesis, identify new therapeutic targets, and develop new interventions. Emerging data identify a key role for chronic inflammation in mediating obesity related disease states and reveal higher incidence of autoimmune disease development. Of the multiple potential mechanisms linking obesity and autoimmunity, the strongest link has been shown for leptin, a hormone secreted at high levels from obese white adipose tissue. Numerous studies have demonstrated that leptin enhances activation of both arms of the immune system, while its absence protects against development of autoimmunity. Other potential newly discovered mechanisms that contribute to autoimmune pathogenesis are not directly connected but also associated with obesity including sustained platelet activation, gut dysbiosis, and aging. Here we review how obesity instigates autoimmunity, particularly in the context of immune cell activations and adipokine secretion.

## Introduction

The world is experiencing an unprecedented rise in obesity–with obesity reaching epidemic proportions in the westernized world. Obesity is defined as a body mass index (BMI >30 kg/m^2^) and is characterized by visceral fat accumulation, subclinical chronic inflammation, and metabolic dysfunction (Cao, 2014). Adipose tissue (AT) dysfunction arising from obesity predisposes affected individuals to multiple sequelae including type 2 diabetes mellitus (T2DM), hypertension, renal, and cardiovascular diseases (Rutkowski et al., 2015). While the connection between obesity and chronic inflammation has been well established (Ellulu et al., 2017), the underlying mechanisms linking obesity-associated inflammation and autoimmune pathogenesis remain unclear. Here we review recent findings that establish stronger links between obesity and autoimmune disease development.

### Changes in white adipose tissue in obesity

Adipose tissue is a specialized connective tissue with adipocytes comprising approximately 35%–70% of its mass (Fruhbeck, 2008). Additional cell types that are present in adipose tissue include epithelial cells, fibroblasts, pre-adipocytes, nerve tissue and immune cells. Two main adipose tissue (AT) types are white adipose (WAT) and brown adipose tissue (BAT). WAT has distinct sites of distribution with the largest stores known as visceral adipose tissue (VAT) in the abdominal area and subcutaneous adipose tissue (SCAT) located under the skin surface and distributed throughout the body.

The onset of obesity changes the normal homeostasis of AT and further alters it’s immune cell content. AT contains innate immune cells (e.g., macrophages, neutrophils, dendritic cells, eosinophils, mast cells, *etc.*) and adaptive immune cells (i.e., T and B lymphocytes). T cells are characterized by their effector functions and are classified by their cell surface molecules, CD4^+^ and CD8^+^. While CD8^+^ T cells primarily function to kill infected cells, CD4^+^ T cells operate as helper (Th) cells that aid in CD8^+^ cell mediated immunity (Th1), B cell maturation and antibody development (Th2), cell mediated inflammation (Th17), and immune tolerance (Treg).

**FIGURE 1:**
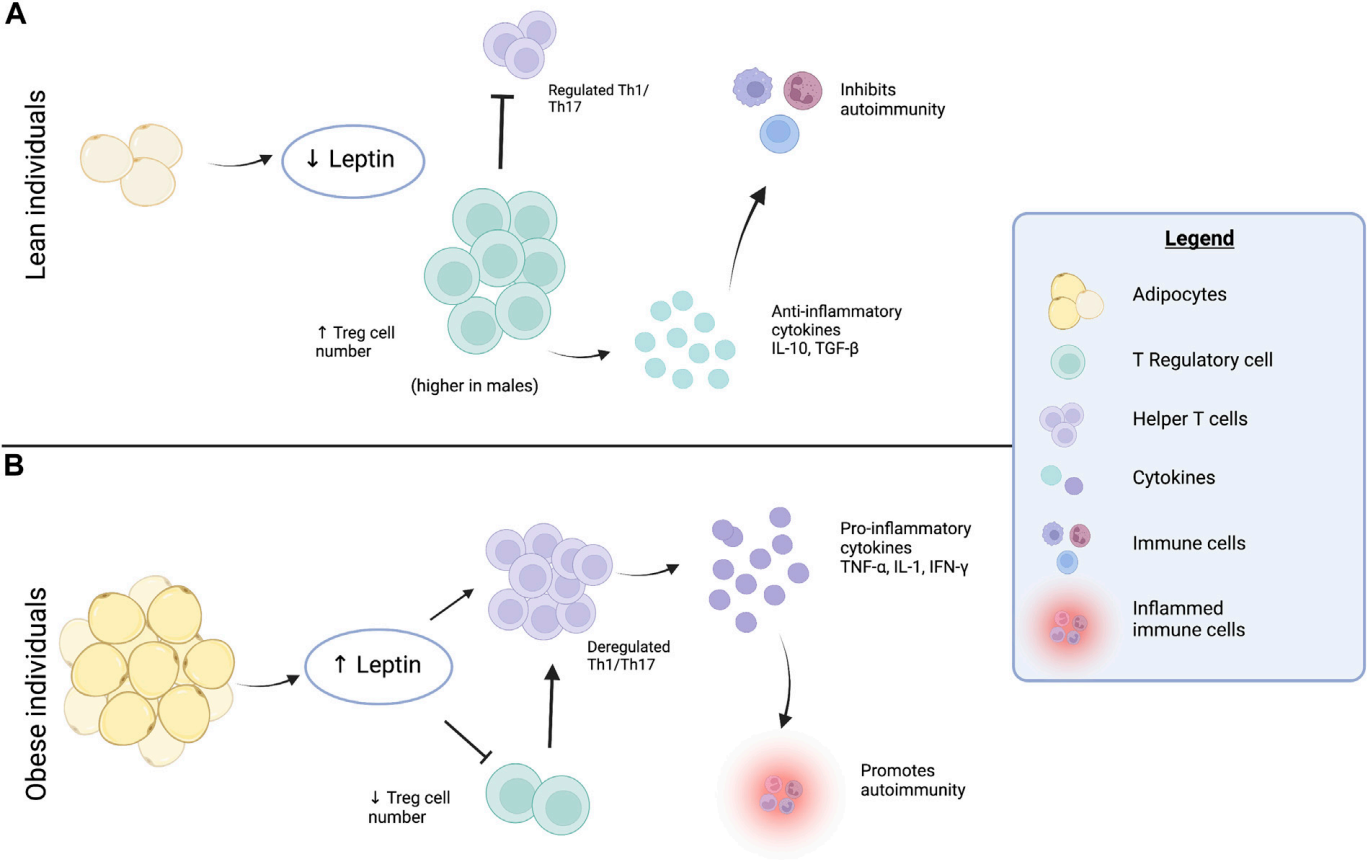
Contributions of the adipokine leptin to adipose tissue inflammation. **(A)** In lean individual’s — adipocytes secrete lower amounts of the adipokine, leptin. Lower levels of leptin are considered protective against pro-inflammatory pathway activation and associated with a regulated Th1/Th17 response and higher numbers of Treg cells in adipose tissue, particularly in males. This leads to secretion of anti-inflammatory cytokines IL-10 and TGF-β, that inhibit and protect against mechanisms that cause autoimmunity. **(B)** In obese individuals — adipocytes secrete higher amounts of leptin, which reduces the number of Treg cells and ultimately causes a dysregulated Th1/Th17 response. The Th1/Th17 cells secrete pro-inflammatory cytokines TNFα, IL-1 and IFN-γ, which promotes autoimmune disease development and dysregulated tissue inflammation. Created with BioRender.com.

In obese AT, a reduction of anti-inflammatory Treg cells is seen–contributing to heightened AT inflammation and insulin resistance. The balance of T cells is shifted toward pro-inflammatory T cell types, which may further recruit circulating monocytes that infiltrate AT. These recruited cells can alter the ratio of macrophage populations in obese AT causing a shift to the pro-inflammatory M1 population and diminution of the anti-inflammatory M2 population (Thomas and Apovian, 2017). Therefore, in obesity the low-grade chronic inflammation of AT is attributed to accumulation of adaptive immune T cells, macrophages, and other immune cells – overall these contribute to increased pro-inflammatory cytokine production (Harman-Boehm et al., 2007; Geeraerts et al., 2017), see Figure 1.

In lean AT, various immune cells secrete anti-inflammatory molecules that promote macrophage type 2 (M2), which have been shown important in preserving insulin sensitivity, see Figure 1. There are important, albeit lesser-known mechanisms necessary for maintenance of WAT that may be disrupted in obesity. This includes a reduction of B regulatory (Breg) lymphocytes (Nishimura et al., 2013), which are most abundant in lean adipose tissue. Breg cells suppress immune responses by secretion of IL-10 and/or TGF-β shown in Figure 1, however, in HFD fed mice CD8^+^ T cells are activated and M1 macrophages accumulate in WAT (Nishimura et al., 2009). The accumulation of pro-inflammatory cells leads to a reduction of Breg cells in WAT– shown to suppress excessive and pathological inflammatory responses, thereby providing protection against autoimmune disease development (Lemoine et al., 2009).

### Triggers of autoimmunity: Activation of self-reactive T cells

Autoimmune diseases are significant clinical issues that are chronic in nature and vary greatly in the organs they impact and their clinical presentation (Rosenblum et al., 2015). Some autoimmune diseases are limited to specific tissues while others disseminate throughout the entire body. Autoimmunity arises as either a combination of genetic predisposing factors or environmental triggers. Documented environmental causes of autoimmunity include climate, hydrocarbon exposure, cigarette smoke, stress, industrial toxins, and diet (Marrie, 2004; Hilgaertner et al., 2011). Autoimmune manifestations typically follow repeating patterns of initiation, propagation, and resolution. These stages are believed to be associated with temporary failure of regulatory immune mechanisms followed by transient or partial restoration of effector and regulatory immune responses. Overall, failure to maintain self-tolerance to self-antigens is the fundamental issue in autoimmunity despite the triggering mechanism (Rosenblum et al., 2015).

The general consensus is that dominant self-tolerance is maintained by Treg cells, which constitute 5%–10% of CD4^+^ T cell populations. Genetic or environmental factors that affect Treg cells can cause or increase susceptibility to autoimmune disease (Wing and Sakaguchi, 2010). Relatedly, depletion of Treg cells can instigate autoimmune disease development in otherwise healthy mice (Sakaguchi, 2005). A key mechanism of Treg cell-mediated suppression of autoimmunity is its control of dendritic cell (DC) functions. Studies centered on understanding the roles of Treg cells in maintaining self-tolerance reveal that Treg cells suppress autoimmune responses but also check other aberrant or excessive immune responses to nonself antigens. It has been shown through immune transfer experiments that the thymus of adult animals normally produces Treg cells but also potentially pathogenic self-reactive T cells. Antigen-introduction into animal models can cause autoimmunity, but at the same time depletion of Treg cells can lead to chronic autoimmune disease that targets multiple organs.

Autoimmunity elicited by Treg depletion does not only occur because a constraint on tissue resident autoimmune T cell clones was removed, but instead Treg depletion also induces activation and expansion of polyclonal T cells as well as maturation of systemic DC populations (Wing and Sakaguchi, 2010). Together Treg cell depletion and subsequent activation of tissue-specific autoimmune T cells contribute to increased DC antigen presentation, higher levels of co-stimulatory molecules, and cytokine release (Kim et al., 2007).

The expansion and differentiation of tissue specific autoimmune T cells influences the numbers of T helper (TH) cell lineages TH1, TH2, and TH-17. In general, mechanisms are in place to preserve peripheral self-tolerance since tissue damage or microbial infections can activate self-antigen presenting DCs that in turn activate pathogenic self-reactive T cells, therefore causing low levels of autoimmunity. In response to inflammation, however, Treg cells are recruited to the tissue site. Within the tissues, Treg cells activate and suppress autoreactive T cell populations, thereby preventing autoimmune reactions from becoming chronic autoimmune disease.

A recent study by Procaccini and colleagues showed that the Treg proliferative response is dependent on cystine/glutamate antiporter solute carrier (SLC)7A11 (Procaccini et al., 2021). *In vitro* studies showed that pseudo starvation of human Treg cells reversed their anergic phenotype. Furthermore, Treg cells obtained from patients with relapsing-remitting multiple sclerosis (RRMS) were found to have reduced proliferative capacity and impaired induction of SL7A11. Patients treated with the immunomodulatory and neuroprotective agent dimethyl fumarate (DMF) had a restored capacity to induce SLC7A11 and regained the capability of expanding Treg cell populations. This study identified a new potential target and enhanced the understanding of metabolic factors that regulate Treg cell proliferation. Treg cells have also been considered “metabolic sensors” that perceive nutritional alterations and modulate AT leptin production and preserve immunotolerance, see De Rosa et al. (2017) for a full review on this topic.

### A role for adipokines

Adipose tissue (AT) serves as an endocrine organ that secretes adipose tissue derived cytokines. Adipokines are cytokines that have important and established roles in metabolism and regulation of energy consumption. More recently, it has been revealed that adipokines contribute to inflammation with potential links to immune dysfunction and autoimmune disease development.

The best characterized adipokines include leptin, adiponectin, resistin, omentin, and vistfarin amongst others (Deng and Scherer, 2010). Leptin is a hormone with key neuroendocrine roles that govern metabolism and food intake (Zhang et al., 1994). This key adipokine is encoded by the LEP gene in humans (ob in mice) and its cognate receptor is LEPR in humans (db in mice). Leptin deficient mice (*ob/ob*) and humans or leptin receptor deficient mice (*db/db*) develop severe obesity (La Cava and Matarese, 2004). However, excessive leptin is secreted by hypertrophic adipocytes upon an increase in fat mass. This paradoxical relationship between leptin and obesity can be explained by development of leptin resistance in obese individuals which become anergic to leptin signaling, resulting in loss of appetite suppression and subsequent weight gain (Tilg and Moschen, 2006). It has been shown that SCAT serves as the major source of leptin in the body (Wajchenberg, 2000).

As mentioned earlier, leptin is not only a mediator of the neuroendocrine system but has also plays a role in immune function (Zhang et al., 1994). The connection between leptin and immune function was recognized in *ob/ob* and *db/db* mice, which were found to have altered immune competence (Chandra and Scrimshaw, 1980). The immunological influence of leptin is not surprising since it is structurally similar to IL-6 (Baumann et al., 1996). Almost all immune cells express leptin receptors (LEPR), and rising leptin levels may contribute to their activation (Francisco et al., 2018). Moreover, it was recently shown that human Treg cells can also produce leptin (De Rosa et al., 2007). The inflammatory action of leptin appears to be that of an acute phase reactant, whereby it causes increased secretion of the pro-inflammatory cytokines IL-6, IL-12, and TNFα (Obradovic et al., 2021). A distinctive sexual dimorphism exists for leptin secretion as sex hormones appear to influence leptin levels. For example, it was found that females secrete higher amounts of leptin than males with equivalent fat masses (Friedman and Halaas, 1998). Meanwhile exposure of AT to pro-inflammatory cytokines IL-1β and TNFα stimulates additional release of leptin from adipose tissue further potentiating circulating leptin levels (Francisco et al., 2018). Additional roles of leptin have been discovered including a critical role in monocyte/macrophage function (Matarese et al., 2005) with the discovery that *db/db* macrophages have impaired killing and phagocytic activity (Dib et al., 2016). Adaptive immune responses are altered by the adipokine leptin as well (La Cava, 2017). Increased leptin levels seen in leptin-competent obese individuals stimulate Th1, Th17 and CD8^+^ T cell responses causing them to dominate while suppressing anti-inflammatory Th2 and Treg cell responses (La Cava, 2017). Overall, when leptin levels are high, Th17 pro-inflammatory functions and B lymphocyte activities are sustained.

Most human studies examining the role of leptin in patients with autoimmune disease have been limited and reserved to extremely rare clinical cases. A patient found to have a unique combinatorial presentation of acquired generalized lipodystrophy and Crohn’s disease (AGLCD) presented with a distinct lack of AT, leptin deficiency (hypoleptinemia), and inflammation of intestinal tissue (Ziegler et al., 2019)*.* Treatment with recombinant N-methylleptin (rLeptin) was initiated to address lipodystrophy in the patient. As a result, the patient exhibited metabolic reprogramming of immune cells, alterations in immune cell differentiation and function, and increased TNFα expression. Ultimately, the increase in TNFα aggravated the Crohn’s disease in the AGLCD patient, however this was attenuated with anti-TNFα based therapeutics (Ziegler et al., 2019).

**FIGURE 2:**
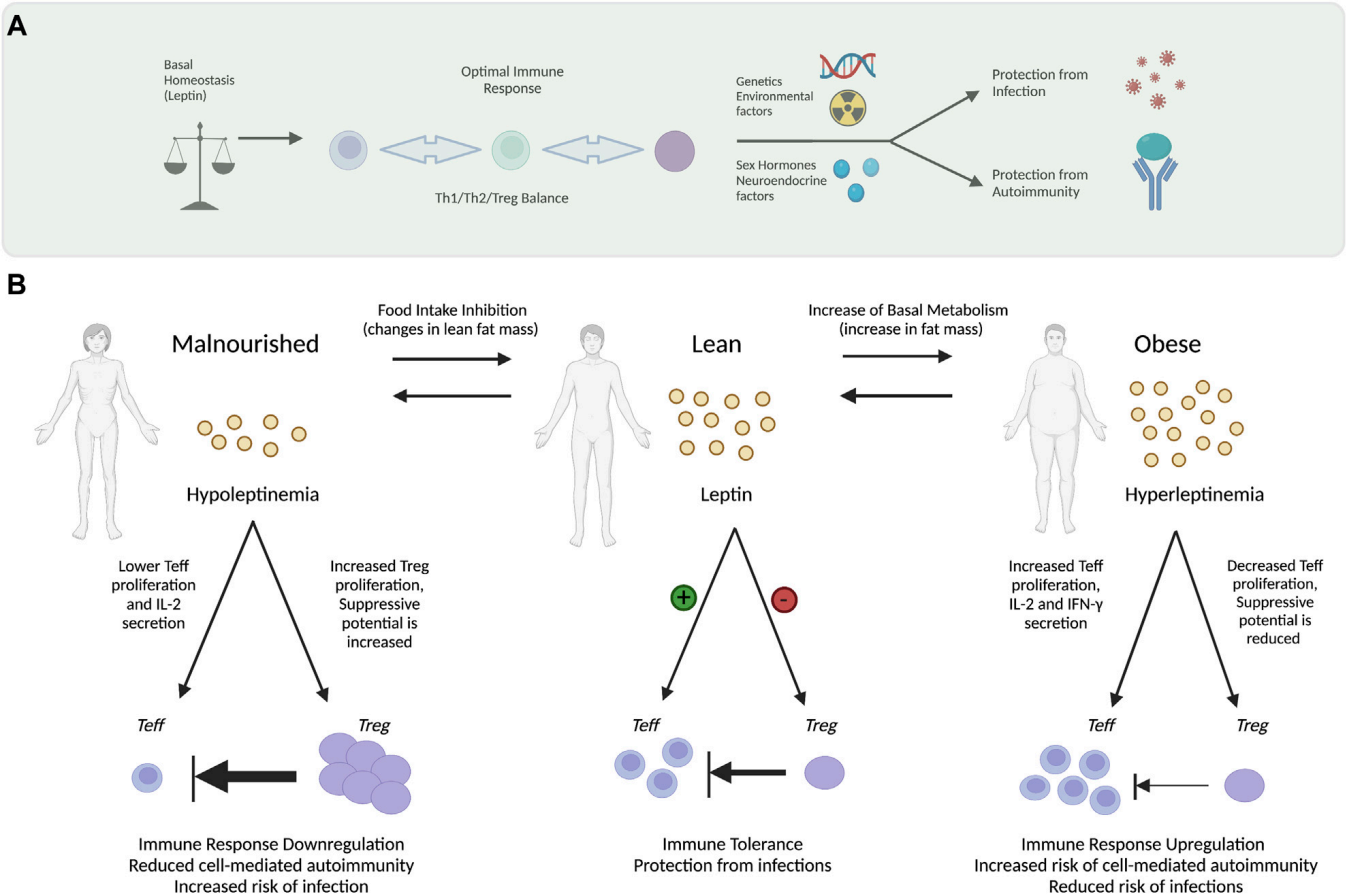
Modulation of Th1 Cell Autoimmunity by Leptin. Leptin serves as a modulator of Th1 cell autoimmunity by its action on effector T cells (Teff) and regulatory T cells (Treg). **(A)** Under basal conditions, homeostatic mechanisms govern leptin levels tuning them for optimal immune responsiveness with the appropriate balance of Th1/Th2/Treg cells that protect from infection but prevents autoimmune disease development. Numerous factors can influence the immune response including genetic factors, environmental triggers that damage DNA, tissue, and immune cells. Neuroendocrine factors such as sex hormones also play an immodulatory role. **(B)** Under homeostatic conditions (center) a lean metabolically healthy individual has relatively low levels of basal leptin secretion by adipocytes. In homeostasis—leptin secreted by adipocytes maintains the Th1 immunity in balance—promoting proliferation of Teff cells while inhibiting expansion of Treg cells. Maintaining the balance between Teff and Treg cells is important for preserving immune tolerance while also protecting against infections. The level of leptin promotes regulated immune function (medium inhibitory arrow) maintaining Treg cell and Teff at steady state levels. Under starvation conditions (left) individuals with substantial weight loss—leptin levels can become too low (hypoleptinemia), which reduces (thick inhibitory arrow) Teff proliferation while increasing Treg cell expansion. Low levels of leptin cause down-regulation of Th1 immunity thereby decreasing the incidence of cell-mediated autoimmune diseases while increasing susceptibility to infections. In obesity (right panel) adipose tissue mass expands and adipocytes express high levels of leptin—subsequently stimulating expansion of Teff cells (thin inhibitory arrow) with a concomitant reduction in Treg cells (thin arrow). Upregulation of immune response increases risk of autoimmune diseases however lowers infection risks. Adapted from Matarese et al. (2008). Figure created using Biorender.com.

Together these findings support the observations that hypoleptinemia (e.g., pseudo starvation, genetic mutation or lipodystrophy) consequently promotes Treg proliferation in humans and mice and protects against immune dysregulation thereby, decreasing susceptibility to autoimmune diseases, whereas conditions that promote hyperleptinemia (e.g., overnutrition and obesity) can instigate immune dysfunction and autoimmunity, see Figure 2.

### Emerging mechanisms linking obesity with autoimmunity

One of the strongest links found thus far between obesity and autoimmunity have come from studies examining the role of leptin on immune function. Earlier studies have shown that chronic leptin- and leptin-receptor deficiency are associated with enhanced infection susceptibility. This included *ob/ob* mice which showed an impaired ability to clear *Klebsiella pneumoniae* bacterial infection (Mancuso et al., 2002). Conversely low leptin levels have been found to protect against autoimmune disease development. Studies in animal models suggest that leptin induces proliferation and polarization of CD4^+^ T helper cells while suppressing development and maintenance of regulatory CD4^+^ T cells (Tregs).

In leptin-deficient *ob/ob* mice, mitogenic stimulation revealed that they have an immunosuppressive phenotype. The *ob/ob* mice exhibited reduced pro-inflammatory cytokine secretion including IFN-γ, TNF, IL-2, and IL-18 with increased production of TH2-type cytokines including IL-4 and IL-10 (Faggioni et al., 2000). The immunosuppression shown in *ob/ob* mice has been found protective against induction of autoimmunity in autoimmune-prone mouse models including experimental autoimmune encephalomyelitis (EAE), which present with demyelination (Sanna et al., 2003; Matarese et al., 2005), experimentally induced glomerulonephritis (EINN) (Tarzi et al., 2004), antigen-induced arthritis (AIA) (Busso et al., 2002), experimentally induced hepatitis (EIH) (Faggioni et al., 2000; Siegmund et al., 2002a) and experimentally induced colitis (EIC) (Siegmund et al., 2002b). In several of these experimental autoimmune studies in *ob/ob* mice administration of leptin restored disease susceptibility to levels of wild-type mice (Faggioni et al., 2000; Matarese et al., 2001; Siegmund et al., 2002a; Siegmund et al., 2002b; Sanna et al., 2003).

Additional studies have examined the impact of defective leptin signaling on a systemic lupus erythematosus prone MRL/Mp-Fas(lpr) mouse model, which spontaneously develops human disease-like lesions, autoantibody production against self-antigens, proliferative glomerulonephritis, and hypocomplementemia (Fujita et al., 2014). The MRL/Mp-Fas(lpr) model lacks Fas protein necessary for apoptotic initiation, is lymphoproliferative and accumulates Treg cells. Blockade of leptin signaling by crossing MRL/Mp-Fas(lpr) mice with C57BL/6J-*ob/ob* mice was sufficient to reduce splenomegaly, decrease the abundance of double negative (CD4^−^, CD8^−^) T cell found in MRL/Mp-Fas(lpr) mice. Examination of serum from these mice revealed lower levels of anti-dsDNA autoantibodies and normal renal histology. According to these findings SLE patients may experience therapeutic benefit upon leptin signaling blockade. The link between circulating leptin levels and the autoimmune disease systemic lupus erythematosus (SLE) was further strengthened by a study by Lourenco and colleagues in 2016 that examined the susceptibility of (*ob/ob*) mice to lupus manifestations and/or SLE disease compared to leptin sufficient wild-type mice. The lupus-inducing agent pristane was used to trigger autoimmunity in leptin-deficient (*ob/ob*) mice. These mice were protected from renal disease, autoantibody production, and had increased Treg cell counts compared to WT. These findings were confirmed in the spontaneous SLE mouse model the New Zealand Black (NZB) Å∼ New Zealand White (NZW)F1 (NZB/W), which had elevated leptin levels that correlated with severity of disease manifestations. For example, administration of leptin further increased autoantibody and renal disease development while leptin antagonism delayed disease and increased survival. *In vitro* studies showed that leptin promoted effector T-cell responses by increasing presentation of self-antigens to T cells. These studies showed that leptin promoted CD4^+^ Treg cell activity, supporting the earlier findings connecting leptin and immunity.

In humans, leptin has been found expressed by T cells and macrophages within lymph nodes and inflammatory lesions of the central nervous system (CNS) in acute and relapsing EAE. Patients with active multiple sclerosis (MS) have increased leptin expression in inflammatory CNS lesions (Sanna et al., 2003) and serum prior to relapse (Lock et al., 2002). De Rosa and colleagues reported that freshly isolated human Treg cells express high levels of leptin and leptin receptors under non-activated conditions. It was also shown that the leptin pathway exerts a negative influence on Treg cell proliferation (De Rosa et al., 2007). *In vitro* neutralization experiments showed that the human Treg cells when treated with anti-leptin monoclonal antibodies had increased proliferation under stimulatory conditions. Relatedly, leptin- or leptin receptor-deficient mice exhibit enhanced Treg cell proliferation, suggesting that leptin and the leptin receptor are negative signals that suppress Treg proliferation. These studies helped clarify findings from earlier investigations that reported that chronic leptin or leptin receptor deficiency increased susceptibility to infection and resistance to autoimmune disease (Farooqi et al., 2002; La Cava and Matarese, 2004; Ikejima et al., 2005).

A rare subpopulation of dendritic cells rich in perforin-containing granules (perf-DCs) have been discovered that may represent a regulatory link between immunological mechanisms that govern adipose tissue metabolism (Zlotnikov-Klionsky et al., 2015)*.* Initial *ex vivo* studies suggested that perf-DCs may have a tolerogenic role (Stary et al., 2007). *In vivo* analyses demonstrated that selective deletion of perf-DCs in radiation-treated chimeric mice caused the mice to develop metabolic syndrome–a phenotype completely prevented by *in vivo* T cell depletion. Additional studies demonstrated that the perf-DCs maintain inflammatory T cells in WAT at steady state, thereby controlling AT inflammation (Zlotnikov-Klionsky et al., 2015). When perf-DC ablated mice were administered a high-fat diet they exhibited an exacerbated metabolic phenotype. These findings were further corroborated in the MS model, experimental autoimmune encephalomyelitis (EAE) where mice lacking perf-DCs exhibited significantly increased autoimmune-associated T cell clones compared to controls. These findings illustrate that minor subpopulations of myeloid DCs exist that may provide clues as to how immune dysregulation accompanies metabolic dysfunction and contribute to the pathogenesis of autoimmunity.

### Emerging autoimmunity triggering mechanisms–Unproven links to obesity

Several notable studies have uncovered new autoimmunity-triggering mechanisms that have not yet been studied in the context of obesity but have their own independent links to obesity including: 1) sustained platelet activation in obese females, 2) translocation of gut pathobionts, and 3) aging related alterations in immune function. A few intriguing potential connections are highlighted below. It has been shown that android obesity in women is linked to enhanced lipid peroxidation and persistent platelet activation (Davi et al., 2002). More recently it has been shown that platelet activation may play a role in the pathogenesis of SLE (Duffau et al., 2010).

Recent studies have shown that translocation of gut pathobionts from the gastrointestinal (GI) tract promotes autoimmunity in genetically susceptible hosts (Manfredo Vieira et al., 2018). Obesity can contribute to gut dysbiosis, which increases the incidence of bacterial leakage into systemic tissues (Cani et al., 2008; Amar et al., 2011; Winer et al., 2016). Aging is associated with increased risk of obesity and immune dysregulation. Whereas the risk of autoimmunity increases with age. Aged individuals have enhanced T cell proliferation and increased DNA in circulation under homeostatic conditions that can activate cytosolic innate immune sensors. The normally nuclear localized, KU complex was found to accumulate in the cytoplasm of aged human and mouse CD4^+^ cells and was shown to mediate a cytosolic DNA-sensing pathway that enhanced T cell activation and the pathology of experimental autoimmune encephalomyelitis (EAE) in aged mice (Wang et al., 2021). As aging increases immune cell dysfunction, age-associated B cells (ABCs) accumulate in autoimmune patients, aged mice, and autoimmune prone mice (Rubtsova et al., 2017) and deletion of the transcription factor T-bet inside B cells prevented autoimmune-prone mice from developing disease. While these findings are not linked to obesity *per se*, they are informative of the mechanisms driving age-related autoimmunity. Additional studies are needed to understand how aging, and obesity intersect.

## Discussion

Obesity related illnesses and comorbidities are on the rise; therefore, it is of fundamental importance that we understand how obesity increases susceptibility for several autoimmune diseases. At present, our understanding of the complex interplay between metabolism and the immune system is that normal metabolic functions have a role in protecting the immune system against aberrant function. The connection between obesity and the pathophysiological mechanisms governing autoimmune disease development has been most thoroughly documented for the adipokine leptin. Studies of leptin or leptin receptor deficient mouse models have revealed striking protection from autoimmune development in many models of induced autoimmunity. Therefore, it is not surprising that increased leptin secretion by obese adipose tissue distinctly impacts immune cell function and disturbs the normal checks-and-balances that maintain the type and number of Teff cells. Emerging evidence suggests that rare subpopulations of immune cells may play previously unappreciated and distinct roles in protecting metabolic function and subsequently safeguarding from autoimmunity. While many co-morbidities are associated with both obesity and autoimmunity (e.g., aging, gut dysbiosis, enhanced platelet activation)—key pieces of data are needed to firmly connect these *via* a non-leptin dependent mechanism. Future studies may provide insight on the convergence of other lifestyle or environmental factors with obesity that erode the body’s natural protective self-tolerance mechanisms.
